# Cannabinoid-mediated Modulation of Oxidative Stress and Early Inflammatory Response after Hypoxia–Ischemia

**DOI:** 10.3390/ijms21041283

**Published:** 2020-02-14

**Authors:** Daniel Alonso-Alconada, Francisco José Álvarez, Felipe Goñi-de-Cerio, Enrique Hilario, Antonia Álvarez

**Affiliations:** 1 Department of Cell Biology and Histology, School of Medicine and Dentistry, University of the Basque Country (UPV/EHU), 48940 Leioa, Vizcaya, Spain; enrique.hilario@ehu.eus (E.H.); antoniaangeles.alvarez@ehu.eus (A.Á.); 2 Biocruces Bizkaia Health Research Institute, Cruces University Hospital, 48903 Barakaldo, Bizkaia, Spain; franciscojose.alvarezdiaz@osakidetza.eus; 3 GAIKER Centro Tecnológico, Parque Tecnológico, ed. 202, 48230 Zamudio, Bizkaia, Spain; goni@gaiker.es

**Keywords:** Cannabinoid, oxidative stress, inflammation, agonist WIN 55,212-2, hypoxia ischemia, fetal lambs

## Abstract

In the process of neonatal encephalopathy, oxidative stress and neuroinflammation have a prominent role after perinatal asphyxia. With the exception of therapeutic hypothermia, no therapeutic interventions are available in the clinical setting to target either the oxidative stress or inflammation, despite the high prevalence of neurological sequelae of this devastating condition. The endocannabinoid system (ECS), recently recognized as a widespread neuromodulatory system, plays an important role in the development of the central nervous system (CNS). This study aims to evaluate the potential effect of the cannabinoid (CB) agonist WIN 55,212-2 (WIN) on reactive oxygen species (ROS) and early inflammatory cytokine production after hypoxia–ischemia (HI) in fetal lambs. Hypoxic–ischemic animals were subjected to 60 min of HI by partial occlusion of the umbilical cord. A group of lambs received a single dose of 0.01 μg/kg WIN, whereas non-asphyctic animals served as controls. WIN reduced the widespread and notorious increase in inflammatory markers tumor necrosis factor (TNF)-α and interleukin (IL)-1β and IL-6 induced by HI, a modulatory effect not observed for oxidative stress. Our study suggests that treatment with a low dose of WIN can alter the profile of pro-inflammatory cytokines 3 h after HI.

## 1. Introduction

In a recent report showing global, regional, and national causes of under 5 mortality during the last 5 years, Liu et al. [1] brought to light that 45% of the approximately 6 million deaths occurred in the neonatal period. Among these, intrapartum-related events, such as perinatal HI, accounted for 0.7 million deaths. To reduce these devastating numbers, much of the research into mechanisms of brain injury focused on cellular and molecular targets. Oxidative stress and inflammation play a key role in acute disordered brain function known as neonatal encephalopathy leading to a deterioration of neurological outcomes [2,3,4]. These two processes encompass several complex biochemical pathways ranging from the release of free radicals targeting lipids, proteins, and DNA to the activation of glial cells leading to the increased expression of pro-inflammatory cytokines, endothelial adhesion molecules, and leukocyte infiltration, finally resulting in brain damage [3,4]. Using a lamb model of perinatal asphyxia, we previously described that HI triggers a widespread overproduction of reactive oxygen species (ROS) together with an early increase in pro-inflammatory cytokine levels [5]. This rise was linked to cell death, as revealed by a positive correlation of ROS and cytokine levels with apoptotic markers. Despite a variety of drugs and potential neuroprotective assays tested in animal models of perinatal brain injury targeting oxidative stress and neuroinflammation [6], there is none included yet in the clinical management of asphyctic newborns. The endocannabinoid system (ECS) is a prevalent neuromodulatory system composed of cannabinoid (CB) receptors (CBRs), their endogenous lipid-based ligands anandamide and 2-arachidonoylglycerol as well as their synthetic-cum-degradative enzymes. This complex structure plays important roles in the central nervous system participating in its response to different endogenous and environmental insults [7]. Two well-identified CB receptors, whose activation has demonstrated to develop a wide range of physiological and disease processes, include the CB type 1 receptor (CB1R) and the CB type 2 receptor (CB2R).

CBRs can be activated not only by their endogenous ligands anandamide and 2-arachidonoylglycerol but also by phytocannabinoids and synthetic CBs. WIN 55,212-2 (WIN) is a potent CB1R/CB2R mixed synthetic agonist with a slightly higher CB2R selectivity compared to other mixed agonists [8]. Our results suggest that the administration of WIN after perinatal asphyxia in fetal lambs reduced brain injury, decreasing both delayed cell death and glial damage [9], together with the maintenance of mitochondrial integrity and functionality [10]. 

However, the potential therapeutic effect of this and other CBs on ischemic disorders is far from clear. CB1R activation can develop either protective or toxic responses after brain ischemia [11], as these receptors can either promote the suppression of glutamate (inducing neuroprotection) or the release of gamma-aminobutyric acid (thus amplifying the toxic response) leading to oxidative stress. In a recent report by Rivers-Auty et al. [12], the CB2R-selective agonist GW405833 did not show a beneficial effect in a model of cerebral HI, despite that CB2R-induced neuroprotection was always related to its anti-inflammatory capacity [13,14]. Thus, the anti-oxidant capacity and/or the anti-inflammatory effect developed by the endocannabinoid system after perinatal asphyxia remain a subject of investigation. This study aimed to analyze the modulatory effect of the CB agonist WIN on ROS and early inflammatory cytokine production after HI in fetal lambs. 

## 2. Results

Physiological parameters from the three groups of lambs included in this work are presented in Supplementary Materials. At baseline, none of the parameters evaluated (pH, paCO_2_, paO_2_, mean blood pressure, base excess, and heart rate) showed significant variations between groups. During the hypoxic–ischemic procedure, HI induced a decrease in pH in both vehicle and WIN-treated animals and recovered after the insult. Delivery (at 0 h) induced temporal acidosis in sham animals, whose pH returned gradually to baseline values during the next 3 h. HI induced a decrease in paCO_2_ and base excess levels, together with tachycardia and an increase in the mean blood pressure. With the exception of the heart rate, these parameters showed no differences between groups during the rest of the experimental procedure. There were no differences in base excess levels at HI between vehicle and WIN-treated animals. Vehicle-treated animals maintained tachycardia and lower levels of paO_2_ during the entire experiment when compared with sham and WIN-treated lambs.

**Figure 1:**
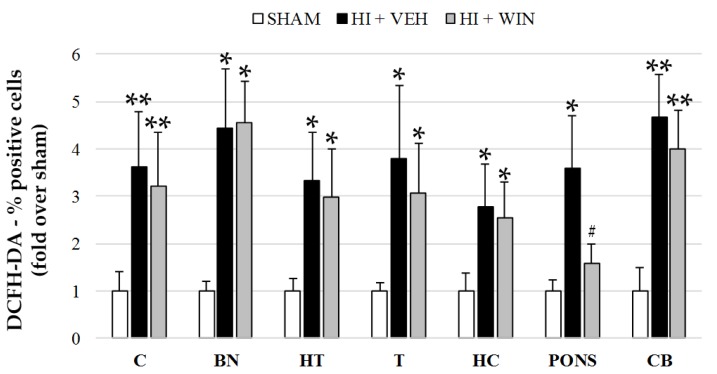
Quantification of ROS in different brain regions evaluated after sham or HI (and vehicle or WIN treatment) in fetal lambs. Samples from both hemispheres were analyzed in triplicate by flow cytometry (*n* = 6) for each animal and condition. Values given as the mean ± SD. (*) ANOVA *p* < 0.05 vs. sham and HI+WIN; (**) ANOVA *p* < 0.01 vs. sham and HI+WIN; (#) ANOVA *p* < 0.05 vs. HI+VEH. Sham, *n* = 6; HI+VEH, *n* = 6; HI+WIN, *n* = 6. (C: cortex; BN: basal nuclei; HT: hypothalamus; T: thalamus; HC: hippocampus; CB: cerebellum).

**Figure 2:**
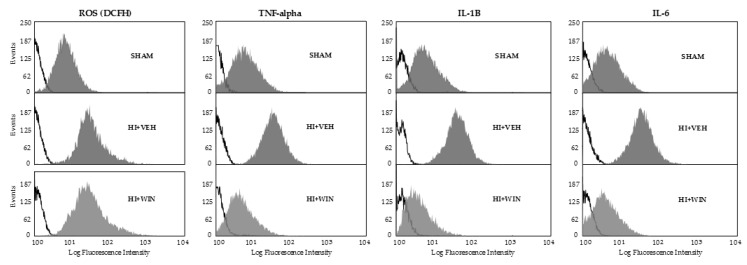
Representative fluorograms obtained after flow cytometry analysis showing ROS, TNF-α, IL-1β, and IL-6 fluorescence. Samples of different brain regions from both hemispheres were analyzed in triplicate by flow cytometry (*n* = 6) for each animal and condition. Black lines correspond to auto-fluorescence, whereas grey areas correspond to positive cells. Sham, *n* = 6; HI+VEH, *n* = 6; HI+WIN, *n* = 6.

Figure 1 shows that ROS evaluation using the fluorochrome DCFH-DA revealed a significant (* *p* < 0.05 or ** *p* < 0.01 vs. sham) increase in its production after HI in all brain regions studied. While the percentage of positive cells for sham animals never exceeded 14.8%, HI raised these values to 53.8% for the cortex, with the rest of the brain regions ranging from 39.1 to 49.9%. Comparing HI with sham animals, this implies an increase of 3 to 5 times (Figure 1 and Figure 2). The administration of the CB agonist WIN solely reduced its production in pons (#*p* < 0.05 vs. HI+VEH), remaining elevated in the cortex, basal nuclei, hypothalamus, thalamus, hippocampus, and cerebellum.

**Figure 3:**
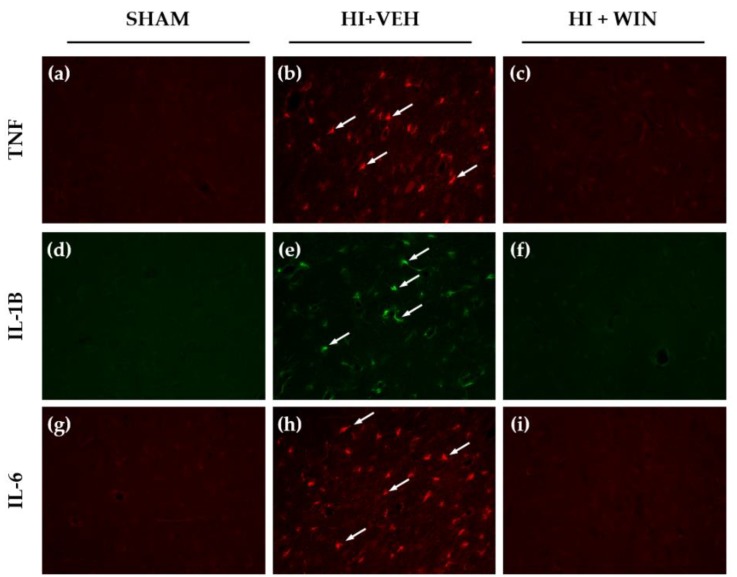
Representative microphotographs 3 h after HI TNF-α (**a**,**b**,**c**), IL-1β (**d**,**e**,**f**), and IL-6 (**g**,**h**,**i**) positive cells after immunofluorescence labeling. Cortex brain sections were obtained after sham operation (**a**,**d**,**g**), HI+vehicle (**b**,**e**,**h**) or HI+WIN treatment (**c**,**f**,**i**). The histological evaluation reveals the presence of these cytokines (arrows) in asphyctic animals. Original magnification: ×200.

Figure 3 shows the histological assessment of the presence of TNF-α, IL-1β and IL-6. Sham brains did not display immunoreactivity for these cytokines (Figure 3a,d,g), whereas TNF-α (Figure 3b), IL-1β (Figure 3e), and IL-6 (Figure 3h) immunoreactivity was markedly increased after HI. Figure 3c,f,i show that animals receiving WIN treatment showed no signs of the presence of these cytokines.

**Figure 4:**
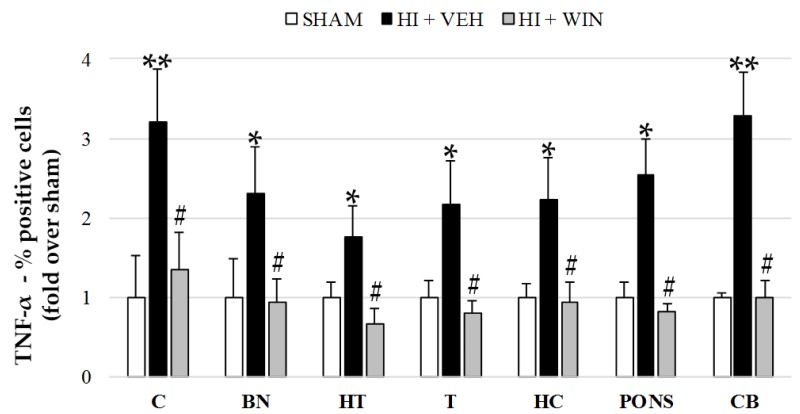
Evaluation of TNF-α expression in different brain regions evaluated after sham or HI (and vehicle or WIN treatment) in fetal lambs. Samples from both hemispheres were analyzed in triplicate by flow cytometry (*n* = 6) for each animal and condition. Values given as the mean ± SD of the percentage of TNF-α positive cells. (*) ANOVA *p* < 0.05 vs. sham and HI+WIN; (**) ANOVA *p* < 0.01 vs. sham and HI+WIN; (#) ANOVA *p* < 0.05 vs. HI+VEH. Sham, *n* = 6; HI+VEH, *n* = 6; HI+WIN, *n* = 6. (C: cortex; BN: basal nuclei; HT: hypothalamus; T: thalamus; HC: hippocampus; CB: cerebellum).

Figure 2 and Figure 4 show TNF-α production revealed by means of flow cytometry showing that the global increase in its expression was reduced after WIN administration. TNF-α levels for both sham and HI+WIN groups ranged between 6.5 and 9.4% for all brain regions; hypoxic–ischemic animals reached values of 21.74% positive cells for basal nuclei (* *p* < 0.05 vs. sham) or 24.13% for cerebellum (** *p* < 0.01 vs. sham). Globally, we showed a twofold increase for brain regions such as the hypothalamus, thalamus, and hippocampus and a threefold increase for the cortex and cerebellum (Figure 4), which decreased after WIN administration (#*p* < 0.05 vs. HI+VEH).

**Figure 5:**
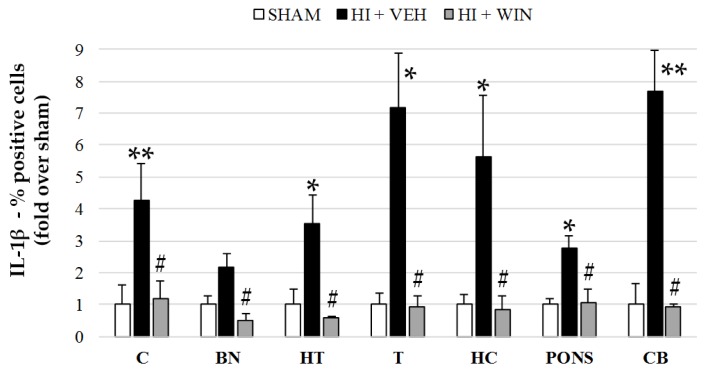
Evaluation of IL-1β expression in different brain regions evaluated after sham or HI (and vehicle or WIN treatment) in fetal lambs. Samples from both hemispheres were analyzed in triplicate by flow cytometry (*n* = 6) for each animal and condition. Values given as the mean ± SD of the percentage of IL-1β positive cells. (*) ANOVA *p* < 0.05 vs. sham and HI+WIN; (**) ANOVA *p* < 0.01 vs. sham and HI+ WIN; (#) ANOVA *p* < 0.05 vs. HI+VEH. Sham, *n* = 6; HI + VEH, *n*=6; HI+WIN, *n* = 6. (C: cortex; BN: basal nuclei; HT: hypothalamus; T: thalamus; HC: hippocampus; CB: cerebellum).

Figure 2 and Figure 5 show results for IL-1β analysis, where we can observe that non-asphyctic animals showed values ranging from 4.10 to 14.22%. As revealed by the HI group, HI generated a widespread and significant increase in IL-1β expression (** *p* < 0.01 for cortex, thalamus, and cerebellum; * *p* < 0.05 for basal nuclei, hypothalamus, hippocampus, and pons). Cerebral brain regions such as the thalamus and hippocampus showed an increase in IL-1β levels of 6–7 times, an effect completely abolished by WIN (#*p* < 0.05 vs. HI+VEH), displaying values similar to those of the sham group (Figure 5).

**Figure 6:**
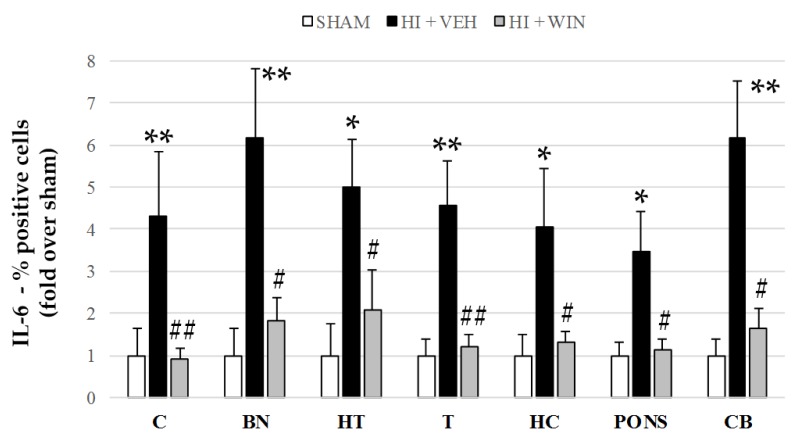
Evaluation of IL-6 expression in different brain regions evaluated after sham or HI (and vehicle or WIN treatment) in fetal lambs. Samples from both hemispheres were analyzed in triplicate by flow cytometry (*n* = 6) for each animal and condition. Values given as the mean ± SD of the percentage of IL-6 positive cells. (*) ANOVA *p* < 0.05 vs. sham and HI+WIN; (**) ANOVA *p* < 0.01 vs. sham and HI+ WIN; (#) ANOVA *p* < 0.05 vs. HI+VEH; (##) ANOVA *p* < 0.01 vs. HI+VEH. Sham, *n* = 6; HI+VEH, *n* = 6; HI+WIN, *n* = 6. (C: cortex; BN: basal nuclei; HT: hypothalamus; T: thalamus; HC: hippocampus; CB: cerebellum).

In the same manner as for TNF-α and IL-1β, but to a lesser extent, Figure 2 and Figure 6 show that IL-6 showed a significant increase in its production after HI in all brain regions studied. The percentage of positive cells for sham animals never exceeds 5%, but HI increased these values to 18.2% for the cerebellum, with the rest of the brain regions ranging from 10 and 15%. This increase was reduced by the administration of the CB agonist WIN (#*p* < 0.05 vs. HI+VEH; (##*p* < 0.01 vs. HI+VEH; Figure 6).

## 3. Discussion

This study evaluated the possible modulation of ROS and pro-inflammatory cytokine production 3 h after a hypoxic–ischemic event in fetal lambs by means of the administration of the CB agonist WIN. This experimental model induced global asphyxia due to umbilical cord compression, reducing blood flow to the fetus, thus affecting the whole brain. In other experimental models, such as the Rice–Vannucci murine model, a lack of tissue irrigation due to arterial blood flow interruption leads to a local pattern of brain injury with damage to the hippocampus, adjacent cortex, and striatum. This study evaluated seven brain regions to obtain deeper knowledge of ROS and the regional expression pattern of cytokine production, analyzing its modulation after CB treatment. The widespread increase in inflammatory markers TNF-α, IL-1β, and IL-6 induced by HI was reduced by WIN, a protective effect not observed when analyzing oxidative stress with the exception of pons.

Early after the initial phase of the acute hypoxic–ischemic insult, a complex and deleterious cascade of events that often result in cell death and tissue damage commences. The lack of ATP obtained from aerobic respiration induces the failure of the Na^+^/K^+^ ATP-dependent pump, leading to neuronal depolarization. This gives rise to toxic accumulation of excitatory amino acids in the synaptic cleft, alongside a huge increase in intracellular levels of calcium, which activates several molecular events, including the production of high levels of toxic ROS, mitochondrial dysfunction, edema, and early cell death [15]. These and other events account for the so-called “acute phase” of brain damage; however, the use of neuroprotective treatments (exception of hypothermia) trying to target these mechanisms has not led to any significant functional improvements, although several clinical trials are ongoing [16]. Later on, oxidative stress and neuroinflammation take the lead in the pathogenesis of brain injury.

Mitochondrial impairment due to membrane depolarization results in a further release of ROS and a decline in endogenous anti-oxidants, generating an imbalance in favor of ROS that can be detrimental for the neonatal brain. Exacerbating ROS generation causes significant damage to biological macromolecules such as proteins (membrane protein degeneration), lipids (lipid oxidation), nucleic acids (DNA degeneration), and other cell constituents, ultimately triggering cell death [17,18]. The antioxidant capacity of the CB agonist WIN has been described both in vitro and in vivo. Oxidative injury in mouse cortical neuron cultures induced through exposure to iron toxicity was reduced after WIN administration, an effect measured by lactate dehydrogenase release and related to reduced neuronal death [19]. Chung et al. [20] determined the effect of WIN on lipopolysaccharide (LPS)-induced ROS production in rat substantia nigra. After WIN treatment, LPS-induced generation of fluorescent products of oxidized hydroethidine was decreased, an effect associated with increased the survival of nigral dopaminergic neurons.

This current work evaluates ROS production in vivo by analyzing DCFH, whose oxidation by ROS transforms this molecule into fluorescent carboxy-DCF and can be measured by flow cytometry. HI caused a generalized increase in ROS production, with values 3 to 5 times higher than in the sham group. However, the CB agonist WIN was not able to ameliorate the widespread production of ROS, with the exception of pons. The lack of an antioxidant effect could be justified in part due to the oxygen concentration used for resuscitation after HI. Even though we have not observed a deleterious role of the use of 100% oxygen ventilation [5,9,10], we cannot rule out a possible harmful effect of this oxygen concentration, an issue observed in the piglet model causing oxidative stress and a dose-dependent oxidation of DNA [21], thus is currently not advised in the clinical situation. On the other hand, we used an ultra-low dose of WIN that, despite showing protective effects in different paradigms of hypoxic–ischemic brain injury [5,9,10], was not enough to activate CB2Rs and was considered to be involved in the antioxidant effects of CBs. WIN showed a higher preference towards CB1Rs (Ki values: 62.3 and 3.3 nM for the human cloned CB1 and CB2 receptors, respectively), whose activation can promote rather than inhibit oxidative stress [22,23]. Several works point out that CB antioxidative signaling is a chemical reaction instead of acting through the CBRs [24,25,26].

Previous studies on CBs and oxidative neuronal injury after neonatal asphyxia have also produced conflicting results. Using a piglet model, Garberg et al. [27], did not find significant differences in neuronal oxidative stress when evaluating urinary levels of neuroprostanes or neurofuranes after treatment with the phytocannabinoid cannabidiol. Pazos et al. [28], however, showed that cannabidiol maintained glutathione/creatine ratio levels similar to those observed in sham piglets after hypoxic–ischemic insult. Glutathione/creatine measurement by proton magnetic resonance spectroscopy is a biomarker that correlates with oxidative stress [29]. The temporal profile of upregulation of ROS, the techniques/markers employed, and the time-points at which evaluations were performed, might be the cause of the discrepancy found in this and previous studies.

Oxidative stress and early inflammatory responses in the developing brain are connected and cannot be considered as separate events. Oxidative stress plays a role in crosstalk between inflammatory systems [17]. Dying and dead neurons activate glial cells to release nitric oxide, a large amount of pro-inflammatory cytokines, and more ROS, with a subsequent direct neurotoxic effect in cell survival. Together with this, permeability of the blood-brain barrier increased by the action of chemokines and matrix metalloproteinases favors the extravasation of blood cells into the brain parenchyma, producing more ROS and pro-inflammatory cytokines, exacerbating inflammation, and resulting in subsequent neuronal death.

Rapid increases in the levels of the main inflammatory cytokines TNF-α, IL-1β, and IL-6 under oxidative stress cause direct injury to the ischemic site [30] and positively correlate with the severity of neonatal encephalopathy [31,32,33,34].

TNF-α plays a pivotal role in the pathogenesis of HI brain injury mediating the initial inflammatory response. In neonatal rats with perinatal anoxia, protein levels of this cytokine were augmented as early as 30 min after the onset of the injury [35], whereas in hypoxic–ischemic animals, both serum and brain-tissue values were significantly increased 4 to 6 h after HI [36]. Acting through its receptor-1, TNF-α can cause both necrosis and apoptosis [37]. In previous work from our group, we showed a positive correlation between the brain region, TNF-α levels, and apoptotic index [5], an association also described by others using murine models of neonatal brain injury [36,38]. In this study, WIN returned HI-induced increases in TNF-α expression to basal values; TNF-α expression levels were twofold or even threefold higher in brain regions such as the cerebral cortex and cerebellum, with respect to the sham group. In previous reports, the ability of WIN to reduce TNF-α responses was demonstrated in LPS-induced bronchopulmonary inflammation [39] and in endotoxemic mice [40]. In our context, using an in vitro model of hypoxic–ischemic brain damage, WIN led to a decrease in TNF-α release in brain slices from newborn rats exposed to oxygen–glucose deprivation, a beneficial effect accompanied by a robust neuroprotective effect [41].

IL-1β is another well-documented pro-inflammatory cytokine involved in the pathogenesis of HI that contributes to neuronal cell death. In experimental models, elevated IL-1β mRNA was detected 3–4 h after hypoxia in neonatal pups [42,43], and, in more recent work, protein expression in the brain cortex was observed 30 min after perinatal anoxia [35]. In a clinical setting, high IL-1β levels in cerebrospinal fluid correlate with the severity of brain injury more than TNF-α or IL-6 and can predict neurological deficits in infants [32]. This implies that IL-1β modulation could reveal therapeutic effects. Significantly, we found that WIN prevented the HI-induced notorious rise of IL-1β expression in lambs: cerebral brain regions such as the thalamus and hippocampus showed an increase in IL-1β levels of 6-7 times, an effect completely abolished by WIN. This blocking capacity of WIN has also been described in rats with chronic cerebral hypoperfusion [44], where the CB agonist downregulated the levels of IL-1β (and of other inflammatory mediators), thus ameliorating neuroinflammation and mitigating chronic ischemic injury. 

Similar to IL-1β and TNF-α, IL-6 increases its expression after HI and can induce aggregation of inflammatory cells in the lesion area, increasing the release of ROS [36,43]. As previously described for TNF-α, IL-6 positively correlates with apoptotic cell death [5,36] and with severity of brain injury in infants suffering from perinatal asphyxia [32]. In addition, WIN reduced the increase of IL-6 levels in all brain regions evaluated after umbilical cord occlusion in fetal lambs. WIN-mediated attenuation of IL-6 expression leading to a decrease in neuroinflammation was also described in aged rats [45] and in an experimental autoimmune encephalomyelitis model mimicking essential pathological features with multiple sclerosis [46].

Despite CBR-independent effects of the compound not being excluded, most neuroprotective effects of CBs in ischemic disorders result from the activation of CB1Rs and/or CB2Rs in neural and immune cells [13,47]. CB2R activation has shown to reduce infarct size after middle cerebral artery occlusion [48] and to decrease inflammation-dependent neurodegeneration, reducing the release of inflammatory cytokines and leukocyte adhesion to cerebral vessels [49]. While CB1R activation mainly regulates the release of glutamate, the synthesis of nitric oxide synthesis, and the accumulation of intracellular calcium [13], we cannot discard an anti-inflammatory role of CB1R. In previous work, the use of selective CB1R antagonists blocked the modulatory effects of WIN on cytokine responses [40,50], indicating that anti-inflammatory effects of WIN can be developed through activation of CB1Rs.

This work has some limitations, such as the use of dimethyl sulfoxide (DMSO) as a vehicle, an excipient not clinically feasible. Despite that DMSO has shown protective effects in some animal models [51,52], other reports suggest the opposite [53], with a lot of discrepancies about the neuroprotective or neurotoxic effects of this compound on the brain. In our work, the dose of DMSO used was lower than that stated to induce neuroprotection [51,52]. On the other hand, if we consider a possible detrimental effect of the solvent, a recent study showed that the negative effect of DMSO in immature rats was prevented by WIN [54]. Here, a direct correlation between cytokine reduction and cell death after WIN treatment was not shown; however, previous reports showed that, after HI, higher levels of cytokines appear in the same brain regions that showed higher levels of apoptotic cell death [5]. Indeed, WIN is able to reduce delayed cell death in fetal lambs [9]. Whether this reduction in the levels of inflammatory mediators developed by WIN (observed 3 h after HI in a model of near-term neonates) can induce long-term neuroprotection or occur in premature or term-born neonates remains unclear.

Accumulating evidence suggests that targeting oxidative and/or inflammatory mechanisms may be a promising avenue for therapeutic intervention after perinatal asphyxia. In the present work, the anti-inflammatory profile (down-regulation of pro-inflammatory cytokines TNFα, IL-1β, and IL-6) described after WIN treatment in fetal lambs is of interest as it demonstrates that CBs can alter the profile of pro-inflammatory cytokines, leading to a reduced level in neuroinflammation.

Further studies are needed to unravel the therapeutic potential of this (and other) treatment(s) for oxidative stress and inflammation after perinatal asphyxia, taking into consideration relevant aspects such as the dose (in this manuscript, only one dose of WIN was tested), the overall impact on the body and long-term outcomes, the temporal schedule of drug administration, and the dual role of some inflammatory mediators such as TNF-α and IL-6 (which can exert either deleterious or beneficial outcomes) or the CBR-dependent and independent effects after CB stimulation.

## 4. Materials and Methods 

### 4.1. Surgery

The experimental model of intrauterine asphyxia of the fetus by means of partial occlusion of the umbilical cord [55] tries to mimic the presence of perinatal asphyxia in a newborn. Lambs were 81%–83% of gestation (120 days), a time-point when animals’ brain maturation reflects the developmental status of a newborn of about 35-36 weeks of gestation [56]. The protocol met the European Union (EU 86/609) and Spanish Government (RD 1201/2005) regulations for animal research, approved by the institutional experimental research committee and has been described elsewhere [5,9,10,55]. Sheep were sedated with 6–8 mg xylazine and 5–10 mg/kg ketamine and were later anesthetized with propofol, 3–4 mg/kg/h, and fetal lambs were exposed to left laparotomy. All animals used in this study received the same anesthesia, including both vehicle and WIN-treated animals.

After exteriorizing the head, lambs were tracheotomized and catheters (XRO umbilical catheter, Vygon, France) were inserted into the right jugular vein to infuse drugs and monitor central venous pressure and into the brachiocephalic trunk through the subclavian artery to measure mean blood pressure (MBP) (OminareCMS24, HP, Göblingen, Germany) and to obtain arterial blood samples. Using a warm blanket (Digiterm S542, JP Selecta, Spain), core temperature was monitored and kept between 37–39 °C throughout the entire experiment, and a non-invasive flow Doppler sensor (T106, Transonics, Ithaca, FL, USA) was used to determine both umbilical venous and arterial blood flows in real time. Two randomizations were performed in blocks of 6 by sealed envelopes: the first block was to randomize the HI or sham procedure. The second block within the HI animals was to randomize the vehicle (HI+VEH) and WIN (HI+WIN) treatments. In lambs assigned to hypoxic–ischemic groups, a vascular occluder was placed around the umbilical cord and compressed to reduce umbilical blood flow to less than 50% of basal value for 60 min. Successful HI was accepted when severe acidosis developed (arterial pH less than 7.1 and base excess to less than −12 mM) [57]. Then, all lambs were extracted by cesarean section, placed on a heater, anesthetized with an i.v. perfusion of ketamine in dextrose 5% (4 mg/kg/h) and paralyzed by a perfusion of atracurium besylate (1 mg/kg/h) and mechanically ventilated for 3 h (Bourns BP200, Riverside, CA, USA). Core temperature was monitored and kept between 37–39 °C by a warm blanket, and parameters such as hematocrit, acid–base balance, gas exchange, mean systemic arterial pressure, heart rate, and carotid blood flow were controlled every 15 min during the entire procedure.

### 4.2. Experimental Groups

Immediately after delivery, HI lambs received (i.v.) a single dose of 0.01 μg/kg WIN (Tocris Bioscience, Bristol, UK) (HI+WIN, *n* = 6) or vehicle (2 mL/kg; Saline + Tween-80 1% + DMSO 2%) (HI+VEH, *n* = 6). Other lambs were similarly managed but without umbilical cord occlusion, serving as controls (sham, *n* = 6). Lambs were maintained, anesthetized, and paralyzed for 3 h until the end of the experiment, when they were euthanized with an intravenous injection of potassium chloride. Brains were flushed with Ringer lactate solution at 4 °C through the carotid arteries, quickly removed and dissected into respective cerebral regions: cerebral cortex, basal nuclei, hypothalamus, thalamus, hippocampus, pons, and cerebellum.

#### Dose Selection

The potential protective effect of this dose of WIN was described earlier [9,10]. Doses of WIN ranging from 100 to 0.01 μg/kg were tested before choosing the lowest one. Higher doses were associated with higher incidence of brain hemorrhages and low mean blood pressure, whereas lower doses showed similar beneficial effects.

### 4.3. In vivo ROS Measurement

Fresh tissue from the seven brain regions evaluated was obtained (double sample), disaggregated (BD Falcon, Becton Dickinson, San Jose, CA, USA), and then labeled with 2′,7′-dichlorohydrofluorescein diacetate (DCFH-DA). Oxidation by ROS causes a further conversion into fluorescent carboxy-2′,7′-dichlorofluorescein (carboxy-DCF). Cell suspensions were incubated with 10 µM DCFH-DA (Molecular Probes, Leiden, The Netherlands) at 37 °C for 30 min. After a loading period of 30 min, cells were washed with Hank’s buffered salt solution, and fluorescence (percentage of positive cells and mean fluorescence intensity) was assessed by an Epics Elite flow cytometer (Coulter Inc., Miami, FL, USA). To exclude debris and cellular aggregates, samples were gated based on light-scattering properties in side scattering (which correlates with cell complexity) and forward scattering (which correlates with cell size). An unstained sample was used as the control to remove the auto-fluorescence. A total of 10,000 events per sample within a gate were collected. 

### 4.4. Immunohistochemistry

Using 4% paraformaldehyde in phosphate-buffered saline (PBS) at 4 °C, brain samples were fixed before cryoprotection by bathing in 30% sucrose. Tissue blocks were then frozen, and 10 µm-thick consecutive coronal sections were cut on a cryostat. Bovine serum albumin was used to block non-specific binding for 1 h at room temperature. Slides were exposed to TNF-α (mouse anti-TNF-α; Serotec, Oxford, UK, 1:50), IL-1β (mouse anti-IL-1β; Serotec, Oxford, UK, 1:50), and IL-6 (mouse anti-IL-6; Serotec, Oxford, UK, 1:50) overnight at 4 °C. After washing with PBS, samples were labelled with Alexa Fluor 488 anti-mouse or anti-goat secondary antibody (1:300, Molecular Probes, Leiden, The Netherlands) for 1 h, followed by several washes with PBS, then dehydrated, mounted and analyzed using a fluorescence microscope. Non-quantitative assessment of staining was carried out on 10 fields of view from each tissue section. All immunostaining for comparison was undertaken in the same experiment. Immunoreactivity was undetectable in negative control samples.

### 4.5. Cytokine Analyses

The same antibodies were employed for the immunohistochemistry assay to perform cytokine analysis. Using the same brain regions evaluated with DCFH-DA, different cell suspensions were collected and labelled with primary antibodies against TNF-α (mouse anti-TNF-α; Serotec, Oxford, UK 1:50), IL-1β (mouse anti-IL-1β; Serotec, Oxford, UK 1:50), and IL-6 (mouse anti-IL-6; Serotec, Oxford, UK 1:50). These antibody concentrations were selected following a series of optimization experiments using a range of antibody dilutions. The experimental protocol followed these steps. A primary antibody was added to each cell suspension that was incubated overnight at 4 °C with 0.5% bovine serum albumin in PBS, and cells were later incubated with Alexa Fluor 488 anti-mouse or anti-goat secondary antibodies (1:300, Molecular Probes, Leiden, The Netherlands) for 1 h. As a control, parallel cell suspensions were processed as above except for the omission of the primary antibody. Samples were numbered, randomly charged in the automatic loader, and the percentage of positive cells and fluorescence intensity were determined by flow cytometry. As earlier reported, samples were gated based on light-scattering properties to exclude debris and cellular aggregates, and 10,000 events per sample within a gate were collected. An unstained sample was used as a control to remove the auto-fluorescence. Fluorescence was estimated at an excitation wavelength of 488 nm and an emission wavelength of 525 nm. All experiments were performed by an investigator blinded to the experimental condition.

### 4.6. Statistical Analyses

In graphs, values are given as the fold-change relative to the sham group ± SD, while some results appear as the mean ± SD. Results were contrasted with Levene’s test to confirm the homogeneity of variance and the Kolmogorov–Smirnov test for normality. Differences between groups in the same brain region were studied by one-factor analysis of variance (ANOVA) with the Bonferroni–Dunn correction. A *p*-value < 0.05 was considered significant. Statistical analysis was performed using SPPS Statistics 24 (SPSS Inc., Chicago, IL, USA).

## Supplementary Materials

Supplementary materials can be found at https://www.mdpi.com/1422-0067/21/4/1283/s1.

## Abbreviations

WIN	WIN55,212-2
HI	Hypoxia–ischemia
CB	Cannabinoid
CB1R	Cannabinoid type 1 receptor
CB2R	Cannabinoid type 1 receptor
CBRs	CB receptors
ROS	Reactive oxygen species
GSH/Cr	Glutathione/Creatine
TNF	Tumor necrosis factor
IL	Interleukin
LPS	Lipopolysaccharide
DMSO	Dimethyl sulfoxide
