# Supplementary material for: Cannabinoid-mediated Modulation of Oxidative Stress and Early Inflammatory Response after Hypoxia–Ischemia
Source: Int J Mol Sci. 2020 Feb 14;21(4):1283. doi: 10.3390/ijms21041283 (PMC7072925; doi:10.3390/ijms21041283)
Supplement: Supplementary file 1 [file ijms-21-01283-s001.pdf]

Supplementary Material

Values of extent of HI and clinical variables of all groups including baseline (before the insult), HI (at the moment the insult was achieved), and from 0h (start of ventilation) until 3h after HI, obtained from [9]. Data are represented as mean (±SEM). Variables: pH (**A**), paCO2 (mm Hg, **B**), paO2 (mm Hg, **C**), MBP (mean blood pressure, mm Hg, **D**), base excess (meq/L, **E**), HR (heart rate, beats per minute, **F**). Sham: sham operated lambs (n=6); HI+VEH: asphyxiated lambs treated with vehicle (n=6); HI+WIN: asphyxiated lambs treated with WIN 0.01 µg/kg i.v. immediately after HI (n=6). * ANOVA, $p<0.05$ vs SHAM.

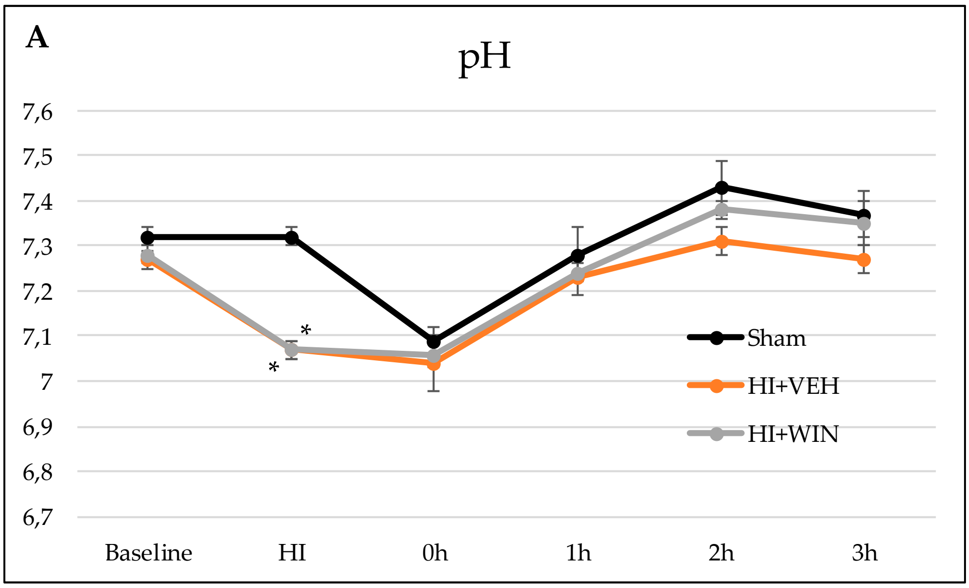

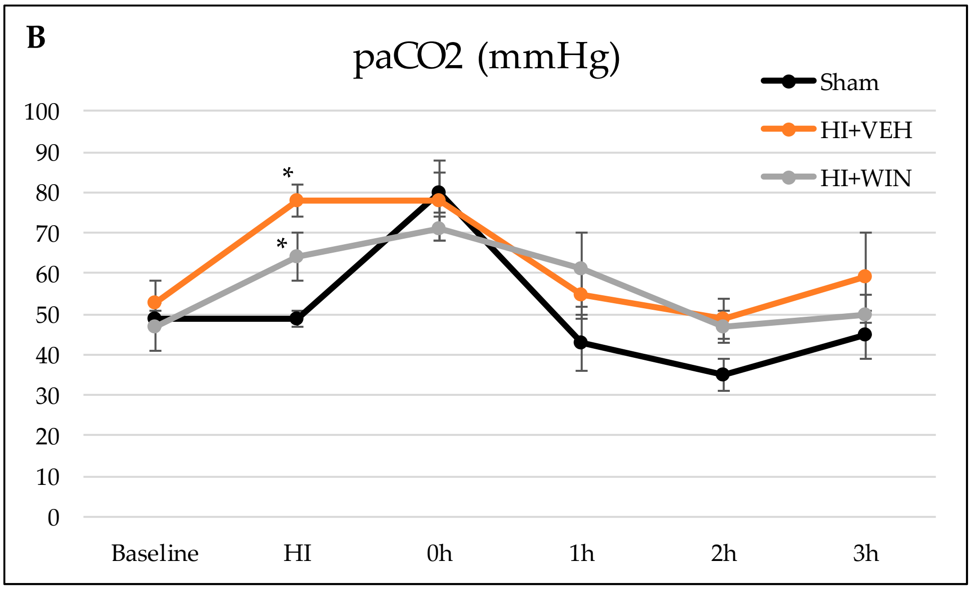

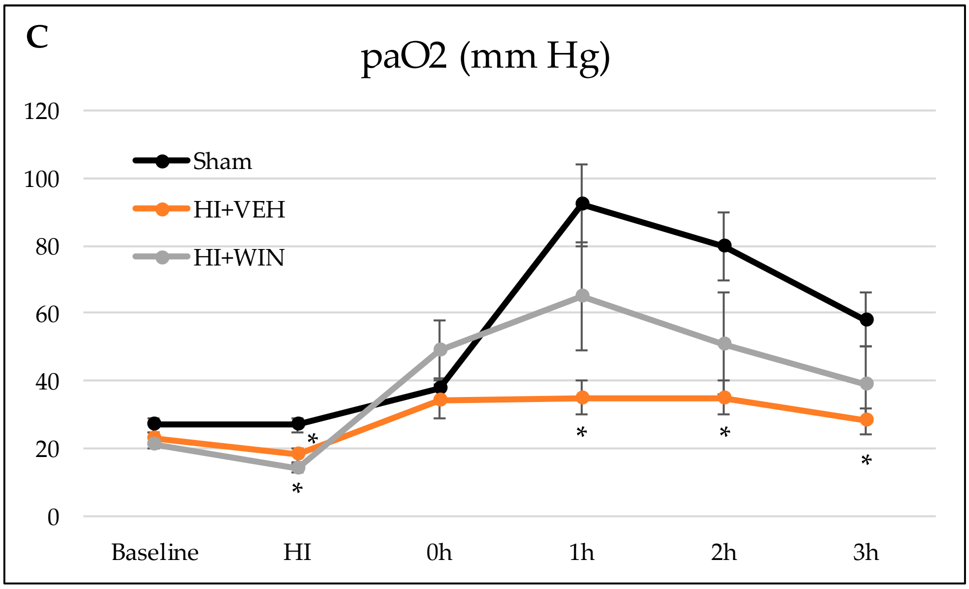
C
paO2 (mm Hg)
120
100
80
60
40
20
0
Sham
HI+VEH
HI+WIN
*
*
*
*
*
Baseline
HI
0h
1h
2h
3h

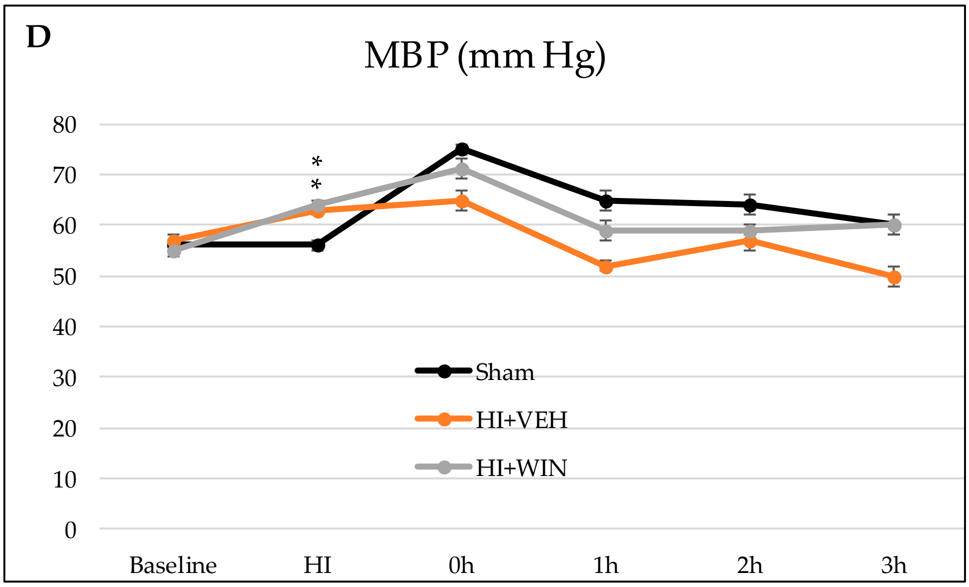
D
MBP (mm Hg)
80
70
60
50
40
30
20
10
0
*
*
Sham
HI+VEH
HI+WIN
Baseline
HI
0h
1h
2h
3h

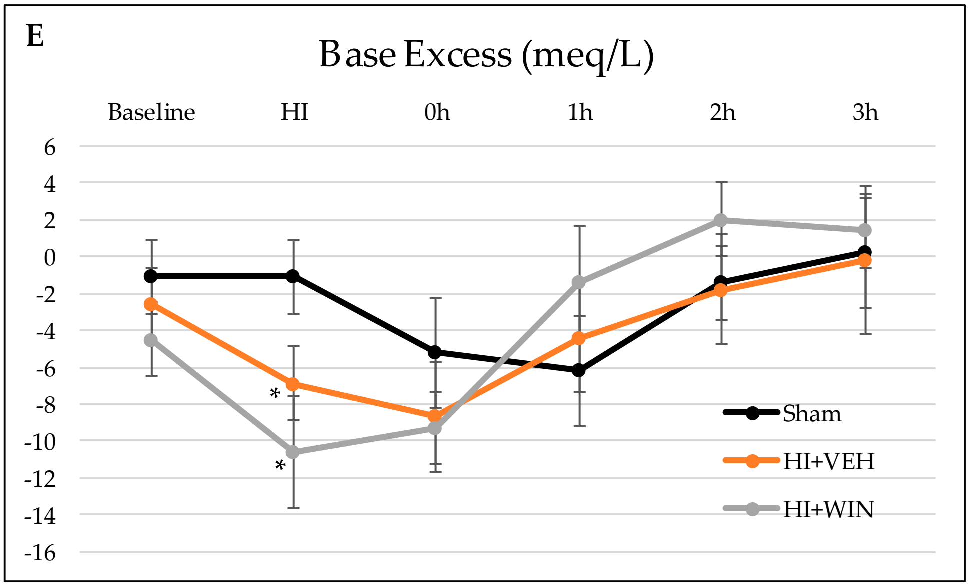
E
Base Excess (meq/L)
Baseline
HI
0h
1h
2h
3h
6
4
2
0
-2
-4
-6
-8
-10
-12
-14
-16
*
*
Sham
HI+VEH
HI+WIN

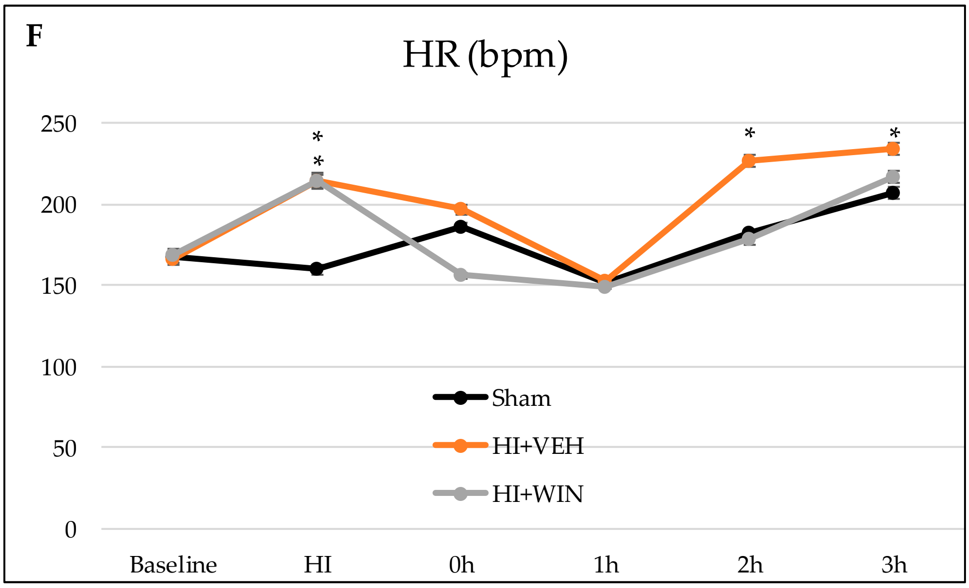
F
HR (bpm)
250
200
150
100
50
0
*
*
*
*
Sham
HI+VEH
HI+WIN
Baseline
HI
0h
1h
2h
3h
